# Enhanced Adsorption of Trivalent Arsenic from Water by Functionalized Diatom Silica Shells

**DOI:** 10.1371/journal.pone.0123395

**Published:** 2015-04-02

**Authors:** Jianying Zhang, Tengda Ding, Zhijian Zhang, Liping Xu, Chunlong Zhang

**Affiliations:** 1 Environmental Science Institute, Zhejiang University, Hangzhou, Zhejiang, People’s Republic of China; 2 Zhejiang Provincial Key Laboratory of Organic Pollution Process and Control, Hangzhou, Zhejiang, People’s Republic of China; 3 Institute of Science and Technology Strategy, Jiangxi Academy of Sciences, Nanchang, Jiangxi, People’s Republic of China; 4 Department of Environmental Sciences, University of Houston-Clear Lake, Houston, Texas, United States of America; University of California, Merced, UNITED STATES

## Abstract

The potential of porous diatom silica shells as a naturally abundant low-cost sorbent for the removal of arsenic in aqueous solutions was investigated in a batch study. The objective of this work was to chemically modify the silica shells of a diatom *Melosira sp*. with bifunctional (thiol and amino) groups to effectively remove arsenic in its toxic As(III) form (arsenite) predominant in the aquatic environment. Sorption experiments with this novel sorbent were conducted under varying conditions of pH, time, dosage, and As(III) concentration. A maximum adsorption capacity of 10.99 mg g^-1^ was achieved within 26 h for a solution containing 12 mg L^-1^ As(III) at pH 4 and sorbent dosage of 2 g L^-1^. The functionalized diatom silica shells had a surface morphological change which was accompanied by increased pore size at the expense of reduced specific surface area and total pore volume. As(III) adsorption was best fitted with the Langmuir-Freundlich model, and the adsorption kinetic data using pore surface diffusion model showed that both the external (film) and internal (intraparticle) diffusion can be rate-determining for As(III) adsorption. Fourier transform infrared spectroscopy (FTIR) indicated that the thiol and amino groups potentially responsible for As(III) adsorption were grafted on the surface of diatom silica shells. X-ray photoelectron spectroscopy (XPS) further verified that this unique sorbent proceeded via a chemisorption mechanism through the exchange between oxygen-containing groups of neutral As(III) and thiol groups, and through the surface complexation between As(III) and protonated nitrogen and hydroxyl groups. Results indicate that this functionalized bioadsorbent with a high As(III) adsorption capacity holds promise for the treatment of As(III) containing wastewater.

## Introduction

Toxic arsenic (As) represents a health crisis for over 100 million people globally, particularly in countries such as Bangladesh, India, and China. Its natural abundance ranks 20^th^, and concentrations as high as 48 mg L^-1^ arsenic were reported in the groundwater in the western US [1–3]. It is, therefore, important to remove arsenic through natural biogeochemical processes or engineered approaches to mitigate its potential environmental and health risk.

The processes or techniques to remove the inorganic trivalent form of arsenite, As(III), is of particular importance because As(III) is 60 times more toxic than the pentavalent arsenate (As(V)) [1]. In aqueous systems, although As(III) is readily oxidized to As(V) under aerobic conditions at pH above 7.0, the trivalent arsenic As(III) exists in its hydrophilic neutral species (H_3_AsO_3_) below pH 9 in anaerobic environments [4]. This neutral species is not amenable to the common removal techniques utilizing anion sorption or anion exchange [5]. Biogenic materials, on the other hand, were reported to be capable of removing As(III) through direct binding with carboxyl and hydroxyl moieties [6]. This bio-based approach has significant biogeochemical implications because it provides a means for the natural attenuation of toxic arsenite in anaerobic environments such as groundwater and the deep aquifer. It also provides an engineered means to remove arsenic through the potential use of biologically derived materials such as biosorbents.

Biosorbents, such as diatom silica frustules, for removing toxic metals and metalloids are highly attractive due to their low-cost and abundant supply from natural biomineralization. Diatoms have been employed as model organisms for the study of nanoscale pattern formation and current materials synthesis approaches due to their intricate 3D hierarchically nanostructured features [7,8]. Various potential applications have been tested such as catalytic support and sorbents. Furthermore, recent studies have revealed the involvement of various functional groups of biological origin (e.g., hydroxyl, amino groups) to bind Hg(II) as well as the strong bonding between thiol (-SH) and Hg(II) [8]. Apart from the high surface area, an added advantage is the high affinity between As(III) and oxyhydroxide through surface complexation. Similarly, fossilized diatoms (or more commonly known as diatomaceous earth, DE) coated with a binary metal (Fe-Mn) oxide provide both oxidation and adsorption capacity, thereby removing As(III) and As(V) simultaneously as was demonstrated in a pilot-scale test [9]. It is thus clear that further research is needed to explore the adsorption of As(III) with diatom silica frustules.

Our approach, presented herein, was to chemically incorporate both thiol (-SH) and amino (-NH_2_) groups into the dried biomass of diatom cells as opposed to introducing a single functional group onto diatomite as has been frequently used in previous studies (e.g., the Hg^2+^ removal using modified DE) [8]. The aim of the present work was to improve arsenic removal through modified diatom frustules and explore their bioadsorption mechanism. For this purpose, silanizing agents with thiol and amino groups were utilized to modify diatom frustules, and the modified sorbents were characterized by scanning electron microscopy (SEM), Brunauer-Emmett-Teller (BET), and X-ray powder diffraction (XRD). The kinetic and thermodynamic parameters of arsenic adsorption were studied in order to gain insight into the adsorption mechanism. The nature of arsenic species on the functionalized diatom frustules was examined by Fourier transform infrared spectroscopy (FTIR) and X-ray photoelectron spectroscopy (XPS).

## Materials and Methods

### Materials

The biomass of *Melosira* sp. was collected from the Freshwater Algae Culture Collection at the Institute of Hydrobiology (FACHB-Collection, Wuhan, China). No specific permissions were required for these activities, and our study did not involve endangered or protected species. The collected diatom samples were washed repeatedly with deionized water to remove surface interfering ions and other undesired materials, such as sand particles and debris. The biomass was then dried in an oven at 60°C for 24 h until constant sample weight was achieved. The dried algae biomass was ground, sieved, and the particles with an average size of 0.45 mm were used for adsorption experiments. Sodium arsenite (NaAsO_2_) was supplied by Shuikoushan Mining Bureau of Hengyang Inc. (China). 3-Mercaptopropyl-trimethoxysilane (MPTMS) and 3-aminopropyl-trimethoxysilane (APTMS) were purchased from Aladdin Chemical Co. (China). All reagents used were of AR grade. High-purity Milli-Q water (resistivity 18.2 MΩ cm^-1^) was used for the preparation of all the aqueous solutions.

### Surface modification of diatom frustules

Diatom frustules were chemically modified with MPTMS and APTMS according to a silanization procedure adapted from Wang et al. [10]. MPTMS is a commonly used coupling agent to incorporate thiol groups (–SH) into the surface of inorganic materials, whereas APTMS has been used to introduce primary amine groups (–NH_2_) to the hydroxyl-bearing silica-based surface. By first adding NH_4_OH in the reaction solution, our modified procedure allowed the hydroxylation to take place prior to the functionalization reactions with both MPTMS and APTMS. Briefly, 0.2 g of diatom frustules was suspended in 20 mL Milli-Q water in a 50 mL conical flask. One mL NH_4_OH (30%) was then added into the mixture and stirred for 2 h at ambient temperature. Then, 1 mL MPTMS and 1 mL APTMS were added into the mixture, which was stirred for 18 h at ambient temperature. After that, the silanized frustules were transferred to a vacuum filtration system, washed with 200 mL Milli-Q water and rinsed with 8 mL ethanol. The frustules were removed from the filtration system, dried in an oven at 110°C for 30 min, and stored in a -20°C freezer prior to use.

### Characterization techniques

The surface morphologies of raw and modified diatom frustules were examined by S3000N scanning electron microscopy (SEM) (Hitachi Co., Japan). FTIR spectra were collected on an Avarat-370 FTIR spectrophotometer (Nicolet Co., USA) using a transmission model. Samples for FTIR determination were ground with spectral grade KBr in an agate mortar. X-ray powder diffraction (XRD) patterns were recorded on a Rigaku D/Max 2550 diffractometer (Rigaku Co., Japan) with a Ni filter and CuKα radiation for crystalline phase identification. The generator voltage and current was 40 kV and 40 mA, respectively. The scan rate was 1° (2θ)/min. X-ray photoelectron spectroscopy (XPS, VG Escalab Mark II, UK) was used to analyze the surface of the samples and examine adsorption mechanisms of the modified adsorbent through electrostatic attraction and covalent bonding. To minimize air contamination to the sample surface, samples were degassed for several hours in the chamber before analysis. The binding energy (BE = 284.6 eV) of the C 1s core level was used as a standard to overcome the charging problem. The XPS spectra were fitted using the Origin 8.5 software, and the composition of the surface layer was determined from the ratio of the relative peak areas corrected by the sensitivity factors of the corresponding elements.

### Batch adsorption procedure

Batch adsorption experiments were carried out in 50 mL flasks at designated pH (2, 4, 5, 7, 9, 10) and Eh values (284, 187, 158, 42, -46, -90 mV). Aliquots containing diatom frustules and arsenite were shaken for the desired contact time on a thermostatic oscillator (HZ-9211K, China) at 200 rpm. The time required for reaching the adsorption equilibrium was estimated by withdrawing samples at regular time intervals until equilibrium was reached. The contents of the flask were centrifugated at 3000 rpm and the supernatant was analyzed for arsenic concentration using an AA6650 atomic absorption spectrometer equipped with HVG-1 hydride generation atomic absorption spectroscopy system (HGAAS, Shimadzu, Japan). A hallow cathode lamp operating at 10 mA was used and a spectral bandwidth of 0.2 nm was selected to isolate the 193.7 nm arsenic line. NaBH_4_ (0.2%) (w/v) in 0.25% (w/v) NaOH was used as a reducing agent, and 5 M HCl was used as the reaction medium. The analytical measurement was based on peak height. Reading time and argon flow rate were selected as 20 s and 200 mL min^-1^, respectively. All batch experiments were carried out in triplicate to obtain reproducibility of the collected data.

### Quality assurance

For analytical calibration, standard solutions with arsenic concentrations ranging from 0 to 0.4 μM As were employed. The limit of detection of the HGAAS was 0.01 μM. Precision of the parallel measurements was ±5% RSD (relative standard deviation). The recovery values for matrix spikes of As(III) and total arsenic were determined in the range of 90–110%.

### Data analysis

Three replicates were included for each treatment of different pH, temperature and arsenite concentrations and the mean ± SD of the sorbed arsenite are presented. The values in enthalpy (ΔH°) and entropy (ΔS°) were acquired through the linear regression between the distribution coefficient (*K*_D_) and temperature (T). The adsorption isotherms were fitted with non-linear regression performed by SigmaPlot 12.0. Nonlinear optimization techniques of error functions were applied to determine best-fit parameters of isotherm and kinetic models (S1 Text).

## Results and Discussion

### Adsorbent characterization

**Fig 1:**
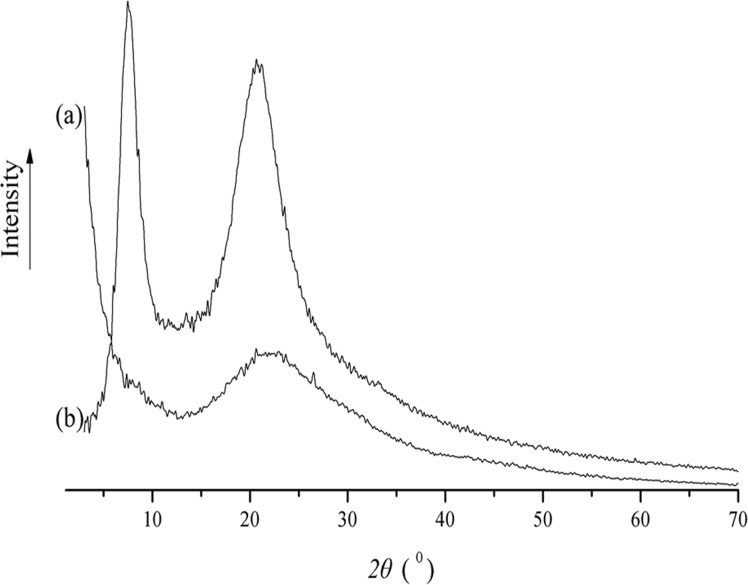
X ray diffraction (XRD) patterns of the raw adsorbent (curve a) and the modified adsorbent (curve b).

The raw and functionalized diatom frustules were subjected to XRD analysis to elucidate the chemical nature of the modification. Contrary to the amorphous raw diatom frustules (curve a in Fig 1), the XRD spectrum for functionalized diatom frustules showed two distinct peaks characteristic of the crystalline presence at 2θ of 7.5 and 21.2°, corresponding to a d-spacing of 11.78 and 4.26 Å, respectively. The peak at 21.2° is attributed to the presence of quartz as previous studies have reported [11,12], whereas the peak at 7.5° can be assigned to the silanizing agent that became incorporated into the modified diatom frustules.

**Fig 2:**
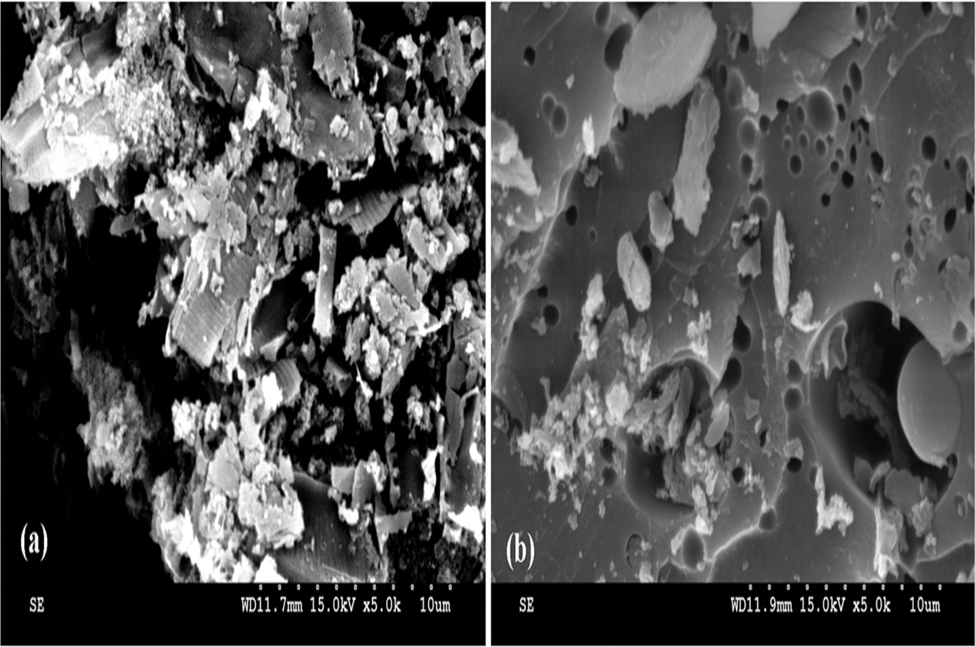
Typical scanning electron micrographs (magnification 5000×): (a) raw diatom frustules, (b) functionalized diatom frustules.

The surface morphological differences between the raw and functionalized diatom frustules were revealed by SEM (Fig 2, S1 Fig). Larger segments and smoother surfaces were observed in functionalized diatom frustules, which may be partially caused by the coupling effect of the silanizing agent. Porous structures were more visible on the functionalized diatom frustules because of the enlarged segments. Additional data from the Brunauer-Emmett-Teller (BET) measurements (S2 Fig, S1 Table) provide supporting evidence for increased pore size at the expense of reduced specific surface area and pore volume as a result of the chemical modification. The reduced surface area and pore volume were likely attributed to the occupation of the porous silica framework by the terminal organic functional groups protruding into the internal surface of the pores as reported by Benhamou et al. [13].

**Fig 3:**
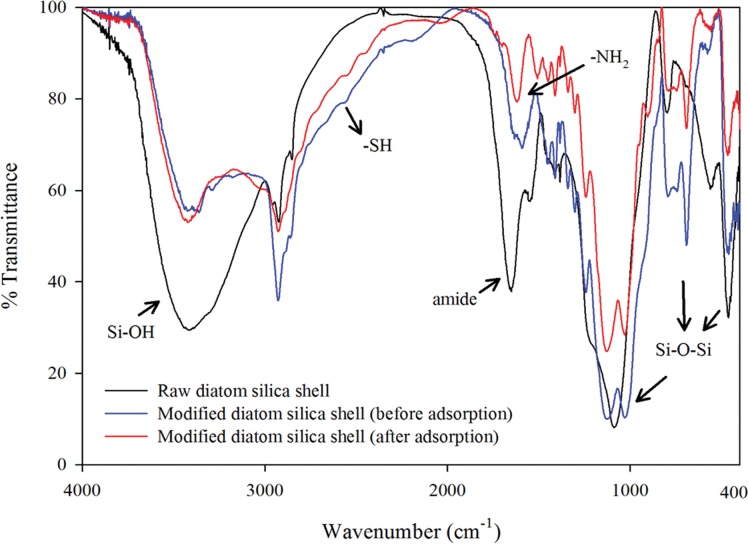
FTIR spectra of raw and modified adsorbents before and after arsenic adsorption.

The incorporation of thiol and amino groups into the diatom frustule structure was confirmed by FTIR (Fig 3). The absorption peaks at 1592 cm^-1^ and 2571 cm^-1^ indicated the presence of—NH_2_ and—SH functional groups on the modified diatom surface [14–16], suggesting the successful grafting of APTMS and MPTMS onto the diatom silica surface. Their stretching frequencies in support of—NH_2_ and—SH functionalization are summarized and compared with the raw diatom silica in S2 Table.

### Effect of solution pH

**Fig 4:**
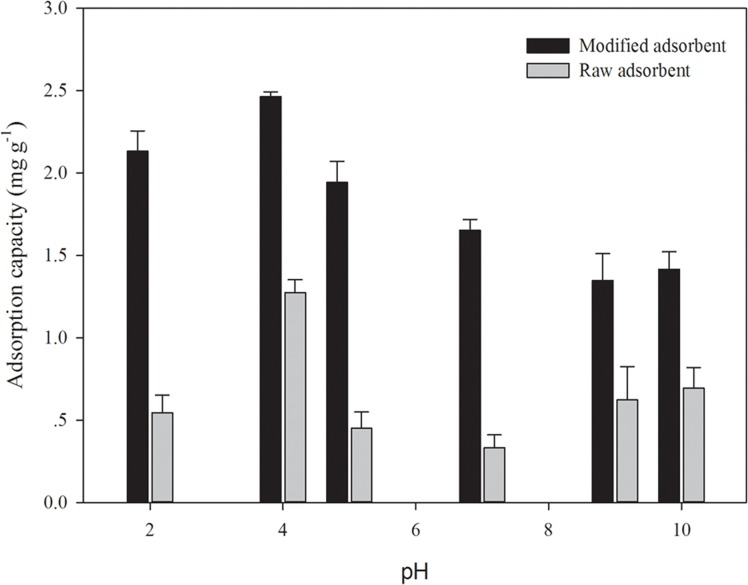
Effects of pH on the adsorption of arsenic (As) by functionalized diatom frustules (As concentration: 160 μM; adsorbent concentration: 1g L^-1^).

The points of zero charge (pH_zpc_) were determined to be 2.47 and 7.40 for raw and modified adsorbent, respectively (S3 Fig). Considering the pH_zpc_ and As(III) behavior, the pH over a wide range of 2 to 10 was selected in this study. In this pH range, an increased pH from 2 to 4 led to enhanced adsorption but beyond this point, increasing pH was not effective. The maximum adsorption efficiency at 1-hr was attained at pH 4 for a solution containing 1 g L^-1^ of modified diatom frustules and 12 mg L^-1^ As(III) at 25°C (Fig 4). Consequently, all the subsequently adsorption experiments were carried out at pH 4. This optimum pH within the acidic range appears to be consistent with the previously reported range (pH 2–6) for optimal arsenic sorption by bacterial biomass and algae [6,17].

The observed pH effects on arsenite sorption in Fig 4 are governed by both the arsenic species in solution and the surface functional groups of adsorbent [18]. In aqueous solution, the predominant As(III) species is the neutral H_3_AsO_3_ at pH < 9.2 (H_3_AsO_3_ = H_2_AsO_3_^-^ + H^+^, pK_a1_ = 9.2), and As(III) in its anionic form (H_2_AsO_3_^-^) becomes important only when pH exceeds 9.2 [1]. The uncharged As(III) species (H_3_AsO_3_) in aqueous medium cannot undergo electrostatic interaction or anionic exchange with the adsorbent, but it can be sorbed through the strong thiol-As bonding at S/As molar ratio of 3 [19]. This thiol-As(III) bonding becomes weakened at alkaline condition (pH > 10) when the predominant H_2_AsO_3_^-^, in lieu of the neutral H_3_AsO_3_, excludes the negatively charged thiolates formed from the dissociation of the thiol group [20]. In addition, previous studies reported high alkaline pH is not favorable for arsenic sorption because carboxyl, hydroxyl, and amide groups of the biomass become negatively charged and a high density of OH^-^ at alkaline conditions would compete with all anionic species of As(III) [11].

### Adsorption isotherms and thermodynamics

**Fig 5:**
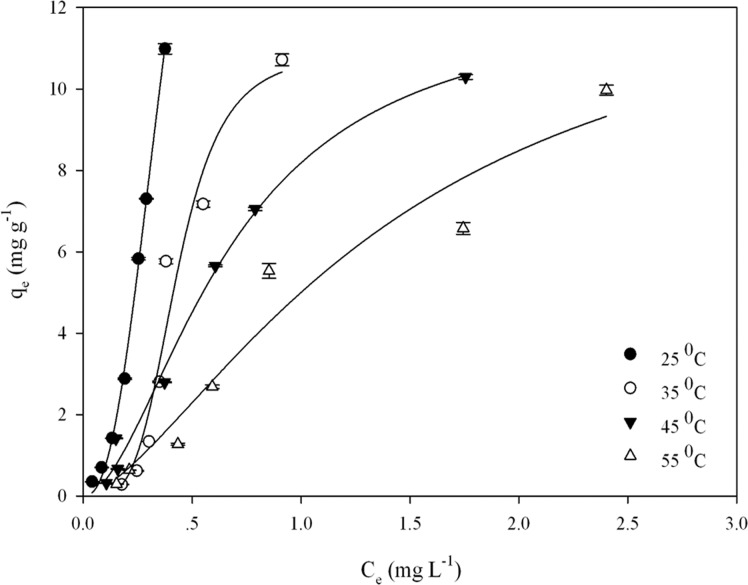
The isotherm plots for the adsorption of arsenic by functionalized diatom frustules at different temperatures (adsorbent concentration: 2 g L^-1^; contact time: 26 h; pH 4).

Adsorption isotherms were obtained with different initial As(III) concentrations ranging from 10 to 300 μM (0.75–22.5 mg L^-1^) as shown in Fig 5. Both two- and three-variable isothermal models including Langmuir, Freundlich, Langmuir-Freundlich, Dubinin-Radushkevich, Redlich-Peterson and Koble-Corrigan [21] were evaluated and the estimated model parameters are listed in Table 1. The best fitted model is deemed to have the highest R^2^ and the least residual sums of square (RSS) values. The Langmuir-Freundlich [22] adsorption isotherm model best fitted the experimental data with the highest R^2^ ranged from 0.949 to 0.998 (p < 0.05) and the lowest RSS values. The maximum adsorption capacity was calculated as 26.13 ± 8.51 mg g^-1^ at 25°C (pH 4). The values of Langmuir-Freundlich constant *K*_LF_ (greater than 1.0 at 25°C) decreased with increasing temperature, suggesting that the adsorption / desorption equilibrium became more reversible and the equilibrium was shifted toward adsorption at the lower temperature [23]. Three different error functions were examined to evaluate the fit of the Langmuir-Freundlich equation to the experimental data (Table 2). Table 2 shows that the lowest value of the sum of the normalized errors (SNE) is obtained using the HYBRID function (25 and 45°C) and an average relative error (ARE) function (35 and 55°C). The parameter values of the Langmuir-Freundlich equation in the corresponding columns are the best-fitted isotherm constants.

**Table 1:** Equilibrium parameters for the adsorption of arsenic onto modified adsorbent^a^.

Model	Parameter values					
	T (°C)	q_m,_ Q_m_,α_R_, a	K_L_,K_F_,K_LF_, K_R_, n	1/n, b, β	R^2^	RSS
Langmuir	25	-8.41±1.69	-1.53±0.18	—	0.980	1.95
	35	-25.43±2.70	-0.33±0.29		0.851	13.69
	45	24.60±1.77	0.43±0.20		0.966	2.85
	55	49.21±1.24	0.10±0.15		0.944	4.42
Freundlich	25	—	66.66±7.38	1.82±0.10	0.995	0.52
	35		12.78±1.88	1.32±0.27	0.871	11.92
	45		7.03±0.50	0.78±0.11	0.944	4.72
	55		4.40±0.49	0.92±0.15	0.942	4.61
Langmuir-Freundlich	25	26.13±8.51	2.35±0.45	2.46±0.30	0.998	0.24
	35	10.90±1.54	2.33±0.25	4.10±1.37	0.951	4.52
	45	12.15±1.08	1.50±0.17	1.81±0.25	0.996	0.61
	55	13.46±8.34	0.71±0.34	1.53±0.78	0.949	4.02
Dubinin-Radushkevich	25	1.05±1.42	—	0.99±0.08	0.967	0.32
	35	62.55±57.16		1.58±0.27	0.876	1.35
	45	0.06±0.10		0.81±0.11	0.919	0.82
	55	0.05±0.05		0.87±0.08	0.961	0.40
Redlich-Peterson	25	-1.10±0.98	1.87±12.26	0.14±1.12	0.986	>100
	35	-1.00±0.08	0.07±0.86	0.01±0.13	0.638	>100
	45	-0.98±2.01	0.14±11.48	0.002±0.17	0.803	>100
	55	-0.98±0.56	0.07±1.55	0.005±0.09	0.887	>100
Koble-Corrigan	25	69.76±55.49	1.94±0.36	-1.52±2.69	0.991	>100
	35	97.58±77.98	3.46±0.63	6.85±8.10	0.931	>100
	45	23.85±9.52	1.84±0.27	1.91±1.23	0.953	>100
	55	6.61±2.23	1.58±0.22	0.44±0.32	0.941	>100

^a^The equations of these models are as follows: Langmuir: *q*_*e*_ = Q_m_K_L_ *C*_*e*_/(1+K_L_ *C*_*e*_); Freundlich:*q*_*e*_ = K_F_ *C*_*e*_^1/n^; Langmuir-Freundlich:q_e_ = Q_m_(K_LF_C_e_)^b^/[1+(K_LF_C_e_)^b^]; Dubinin-Radushkevich: ln*q*_*e*_ = lnq_m_-β*ε* ^2^; Redlich-Peterson: q_e_ = K_R_C_e_/(1+α_R_C_e_^β^); Koble-Corrigan: q_e_ = aC_e_^n^/(1+bC_e_^n^).*Q*_m_is the monolayer adsorption capacity of the sorbent (mg g^-1^); *K*_L_ is the Langmuir adsorption constant (L mg^-1^) related to the free energy of adsorption;*K*_f_ is a constant related to the adsorption capacity, and 1/n is an empirical parameter related to the adsorption intensity which varies with the heterogeneity of the sorbent; *q*_m_ is the maximum adsorption capacity in Dubinin-Radushkevich equation (mol g^-1^), β is the activity coefficient related to the mean free energy of adsorption (mol^2^J^-2^), and ε is the Polanyi potential (ε = RT ln(1+1/*C*_*e*_)). K_LF_ [(L mg^-1^)^1/b^] is the Langmuir-Freundlich constant, and b (dimensionless) is the Langmuir-Freundlich heterogeneity constant, RSS is the residual sums of square. In the Redlich-Peterson isotherm, K_R_ is Redlich–Peterson isotherm constant (L/g), α_R_ is Redlich-Peterson isotherm constant (L/mg) and β is the exponent which lies between 0 and 1. In the Koble-Corrigan model, a, b and n are the Koble–Corrigan parameters.

**Table 2:** Langmuir-Freundlich parameters with error analysis/error function.

Parameters	Hybrid fractional error (HYBRID)	Marquardt’s percentage standard deviation (MPSD)	Average relative error (ARE)
At 25°C:			
Q_m_	7.03	13.55	15.08
K_LF_	0.63	1.22	1.36
b	0.66	1.28	1.42
SNE	**2.49** ^a^	4.80	5.34
At 35°C:			
Q_m_	3.45	6.13	2.25
K_LF_	0.74	1.31	0.48
b	1.30	2.31	0.85
SNE	1.00	1.78	**0.65**
At 45°C:			
Q_m_	2.00	4.92	2.51
K_LF_	**0.25**	0.61	0.31
b	0.30	0.73	0.37
SNE	0.25	0.61	0.31
At 55°C:			
Q_m_	3.00	6.34	2.52
K_LF_	0.16	0.33	0.13
b	0.34	0.72	0.29
SNE	2.10	4.46	**1.78**

^a^ Values in bold represent the minimum sum of normalized errors (SNE).

**Fig 6:**
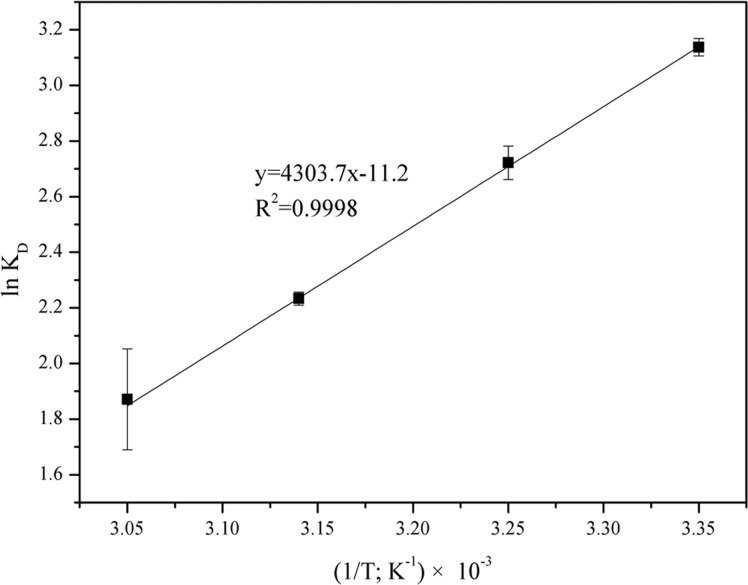
The van’t Hoff plot of lnK_d_ vs. 1/T for the estimation of thermodynamic parameters for arsenic sorption by functionalized diatom frustules (adsorbent concentration: 2 g L^-1^; contact time: 26 h; pH 4).

Several thermodynamic parameters were estimated to provide an insight into the inherent energetic changes in the process of adsorption. The changes in Gibbs free energy (ΔG°), enthalpy (ΔH°) and entropy (ΔS°) are calculated using the following equations [11]:

$$\Delta G^{0}=-RT\ln K_{D}$$ (1)

$$\ln K_{D}=\Delta S^{0}/R-\Delta H^{0}/RT$$ (2)

where R is the universal gas constant (8.314 J mol^-1^ K^-1^), T is temperature (K) and *K*_D_ is the distribution coefficient equating to the ratio of the sorbed arsenic concentration (*q*_e_) to the aqueous phase arsenic concentration (*C*_e_) at equilibrium. The ΔG° values were calculated using the lnK_D_ given in the van’t Hoff plot (Fig 6) and found to be -7.78, -6.97, -5.91, and -5.10 kJ mol^-1^ for temperature at 25, 35, 45, and 55°C, respectively. The negative ΔG° values indicated the spontaneous nature of the adsorption, and the increased ΔG° values with the elevated temperature illustrate a lower adsorption efficiency at higher temperatures. It was reported that ΔG° values up to 20 kJ mol^-1^ were consistent with electrostatic interaction and the typical chemical bonding energy for an ion-exchange mechanism is in the range of 7.9–16 kJ mol^-1^ [24]. The low ΔG° values in this study (< 7.9 kJ mol^-1^) indicate that ion-exchange may not play a significant role in the adsorption processes. Thus, the covalent binding of arsenite to form a surface complex is possibly the major mechanism responsible for the arsenic adsorption process. In addition, the negative value of ΔS° (-92.20 ± 1.42 kJ mol^-1^ K^-1^) reveals the thermodynamically decreased randomness at the solid-solution interface during the adsorption process. Furthermore, the ΔH° was found to be -35.31± 0.44 kJ mol^-1^, indicating the exothermic nature of the adsorption processes at 25–55°C. It was reported that the ΔH° value in the range of 2.1–20.9 kJ mol^-1^ is indicative of physical adsorption, whereas chemical adsorption corresponds to ΔH° in the range from 20.9 to 418.4 kJ mol^-1^ [25]. These results all collectively point to chemisorption as the mechanism primarily responsible for the adsorption of arsenic by modified diatom frustules.

### Adsorption kinetic modeling parameter calculations

As(III) adsorption by modified diatom frustules can be described by the surface pore diffusion model (S1 Text) with two defining parameters, namely the external mass transfer coefficient (β_e_) and the internal (intrapore) diffusion coefficient (D_*p*_). β_e_ describes arsenic transfer through the boundary layer due to concentration gradient from the bulk solution to the surface of adsorbents, which is governed by Fick’s first law. The value of β_e_ can be estimated from the slope of the linear regression (S4 Fig) as shown in (Eq 3) [26].

$$\ln\frac{C}{C_{0}}=-\beta_{e}\frac{Ma_{s}}{V_{L}}t$$ (3)

where C and C_0_ are bulk concentration and initial bulk concentration of arsenite, respectively (mol m^-3^), *β*_*e*_ is external mass transfer coefficient (m s^-1^), M is the mass of the adsorbent (kg), V_L_ is solution volume (dm^3^), *a*_*s*_ is the specific external surface area (m^2^ kg^-1^) as defined by: *a*_*s*_ *=* 6/(*ρ*_*p*_ *d*_*p*_), where *ρ*_*p*_ is the apparent particle density (kg m^-3^) and *d*_*p*_ is mean particle size (m). Using the slope of the linear regression between ln(*C*/*C*_0_) and t (S4 Fig) and the values of other parameters detailed in S1 Text, the *β*_*e*_ value is estimated to be 1.44×10^–6^ m s^-1^ for As(III). The accuracies of this kinetic model (Eq 3) were evaluated by calculating the root mean standard error (RMSE) and average relative error (ARE). The smaller value of RMSE (0.18) than ARE (37.38) indicates that RMSE is adequate to validate this kinetic model.

The internal (intrapore) diffusion coefficient (D_*p*_) is directly proportional to the free diffusivity of arsenic and two parameters related to the porous structure of the diatom frustules that impacts arsenic diffusion in pore water according to (Eq 4) [27]:

$$D_{p}=\frac{\varepsilon_{p}D_{1}}{\tau }$$ (4)

where D_1_ is the free diffusivity for As(III) (11.6×10^–10^ m^2^ s^-1^) [27], *ε*_*p*_ is the particle porosity determined from the BET analysis (*ε*_*p*_ = 0.17), and *τ* is the tortuosity factor which can be calculated from *ε*_*p*_ according to: *τ =* (2-*ε*_*p*_)^2^/*ε*_*p*_. Using values of *ε*_*p*_ (0.17) and *τ*(19.7), the D_p_ is estimated to be 1.0×10^–11^ m^2^ s^-1^.

Furthermore, the dimensionless pore Biot number (Bi_p_) for surface diffusion can be used to evaluate the relative importance of internal (pore) and external mass transport resistances, which can be estimated by (Eq 5) [27]:

$$Bi_{p}=\frac{\beta_{e}d_{p}}{2D_{p}}$$ (5)

Thus a high external mass transfer resistance or low internal mass transfer resistance corresponds to the lower values of *Bi*_*p*_. The estimated *Bi*_*p*_ of 0.06 for As(III) indicates that both the external and internal diffusion were the rate-determining step in the adsorption process [26].

### X-ray Photoelectron Spectroscopy Analysis

The surface elemental compositions of raw and functionalized diatom frustules calculated from XPS data confirmed changes in the atomic concentrations of C, Si, N and S as a result of the surface modification (Table 3). The concentration increases in carbon (56.4% to 60.3%) and silicon (9.1% to 10.7%) in functionalized diatom frustules correspond well to the carbon chains and silicon element introduced from the MPTMS and APTMS. The increased sulfur concentration from 0 to 4.7% in functionalized diatom frustules indicates a successful surface functionalization with thiol groups. However, the decreased concentrations of nitrogen (3.4% to 2.0%) and oxygen (31.1% to 22.3%) indicate that certain nitrogen compounds on the raw diatom frustules may be released into solutions in the modification process, which agrees at least partially with the disappearance of amide bonds observed by FTIR spectrum.

**Table 3:** Elemental compositions obtained from X-ray photoelectron spectroscopy.

Elements	Raw adsorbent (%)	Modified adsorbent (%)	Modified adsorbent with adsorbed arsenic (%)
C	56.4	60.3	65.1
O	31.1	22.3	20.9
Si	9.1	10.7	8.3
S	-	4.7	3.9
N	3.4	2.0	1.5
As	-	-	0.3

**Fig 7:**
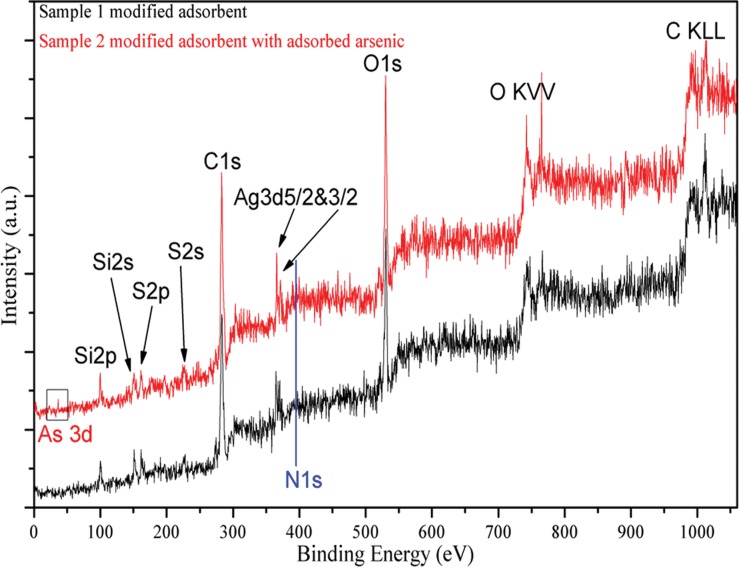
Full-range XPS spectra of modified adsorbent before and after arsenic adsorption.

**Fig 8:**
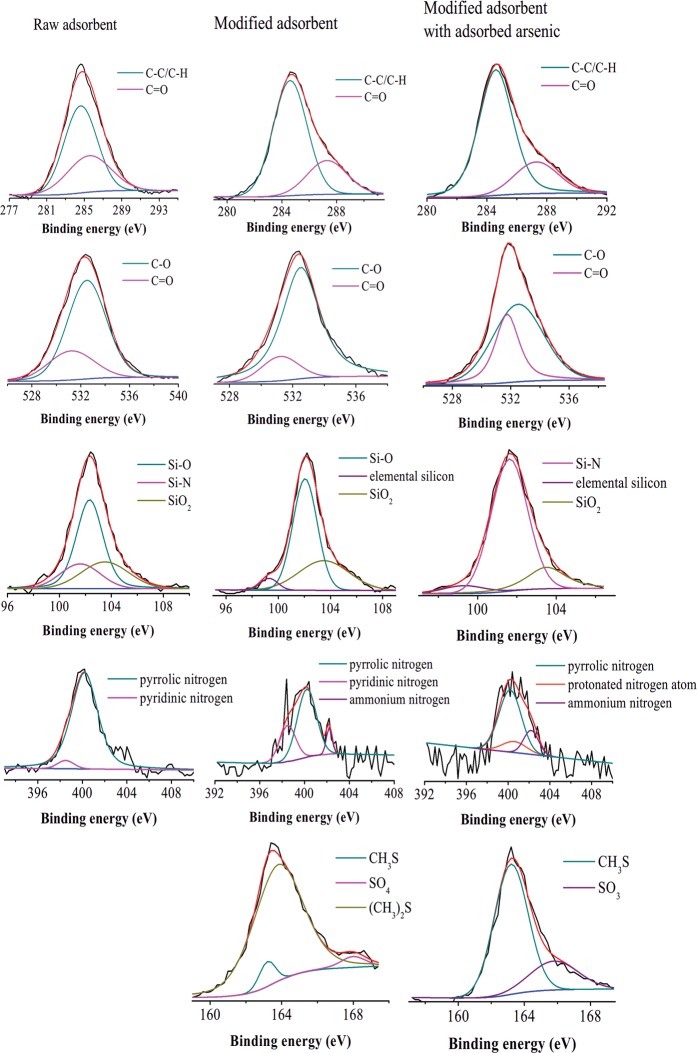
X-ray photoelectron binding energy curves: (a) C1s spectra, (b) O1s spectra, (c) Si2p spectra, (d) N1s spectra, (e) S2p spectra.

XPS can also be used to qualitatively characterize the surface states of modified adsorbent before and after adsorption of arsenic ions. The full-range XPS spectra before and after As adsorption (Fig 7) reveals the presence of arsenic on modified adsorbent, even though some fluctuations were observed on the As 3d spectrum due to a small amount of arsenic detected on the surface. The observed entry of arsenic into pores on the adsorbent is in agreement with the results of the mass transfer model. XPS spectra of the other-related elements (Fig 8) were further analyzed to facilitate the mechanistic understanding of arsenic adsorption on diatom frustules. Note that the high-resolution XPS spectra were analyzed using a curve-fitting procedure based on the Gaussian-Lorentzian function after baseline subtraction using Shirley’s method.

The deconvolution of C 1s bands allowed quantification of the spectral contribution of two carbon species assigned to C-C/C-H and C = O species in raw and modified adsorbent, and correspondingly yielded two Gaussian peaks at 284.6 eV and 287.3 eV, respectively (Fig 8). The chemical modification increased the percentage of C-C/C-H due to the introduction of carbon chains from MPTMS and APTMS into the surface. The O 1s spectra of adsorbents were obtained through curve-fitting of two peaks at 531.2 eV (C = O) and 532.5 eV (C-O), respectively, and the percentage of C = O increased. After adsorption, the percentage of C = O declined, but C-O contents increased compared to the modified adsorbents, indicating the carboxyl groups may have participated in the adsorption process that caused the rupture of the double bond in C = O, and subsequently formed arsenic oxides (As_2_O_3_) with arsenic ions. Prior studies also quantified the spectral contribution [28,29], and indicated the formation of As-O can become an important mechanism for the adsorption of organic arsenic [30,31]. In addition, the Si 2p peaks indicate three different chemical states of silicon. The peak at a binding energy of 101.6 eV and 102.5 eV in the raw adsorbent can be attributed to Si-N and Si-O [32,33], and the peak observed at 103.5 eV is due to SiO_2_ (Si^4+^) [34]. The chemical modification destroys Si-N, and a new peak at a binding energy of 99.3 eV is formed due to elemental silicon (Si°) [35]. The disappearance of Si-O subsequent to adsorption indicated that Si-O was involved in the adsorption of arsenic, which is consistent with FTIR results.

Analysis of the N 1s peaks for modified adsorbent (Fig 8) reveals three peaks showing the presence of nitrogen in three different environments. The peaks centered at 398.5 eV, 400.2 eV, and 402.2 eV correspond to pyridinic nitrogen, pyrrolic nitrogen, and ammonium nitrogen, respectively [34]. The appearance of the ammonium nitrogen peak compared to the raw adsorbent indicates the protonation of some of amine groups. However, when arsenic was adsorbed on the modified adsorbent, a clearly higher binding energy peak at 400.6 eV was noted, which was attributed to the protonated nitrogen atom (N^+^). The increased proportion of this positively charged nitrogen atom indicated that the protonated nitrogen atom may have reacted with arsenite through surface complexation. Meantime, the FTIR spectra of the adsorbent after arsenic adsorption, as shown in Fig 3, revealed the shift of stretching bands of the—NH_2_ group and—NH_3_^+^ group to 1619 cm^-1^, which was also indicative of the involvement of protonated amines in adsorption.

The successful introduction of the sulfur element in the modified adsorbent can be seen in the S 2p spectra (Fig 8). It reveals three peaks in the modified adsorbent located at163.2 eV, 163.8 eV, and 168.0 eV, which can be attributed to chemisorption as a result of the covalent bonding between thiols and neutral As(III) species. These three peaks correspond to methanethiolate (CH_3_S-), chemisorbed dimethyl sulfide ((CH_3_)_2_S) and sulfate (SO_4_^2-^) [36]. Since the intensity of the (CH_3_)_2_S peak became weaker than the CH_3_S peak after As adsorption, the methyl groups might exchange with arsenic. For the SO_4_^2-^ peak, the binding energy was shifted to a lower energy at 165.8 eV that belongs to SO_3_ after As adsorption [37]. The omitted oxygen may be transferred by forming As-O with arsenic ions. Hao et al. [20] reported that each As(III) atom in H_3_AsO_3_ can bind three sulfur atoms, indicating As^3+^ was present on the adsorbent surface. The stretching bands of—SH group in the FTIR spectra were shifted to approximately 2559 cm^-1^ with a marked change in the transmittance. The peak 791 cm^-1^ appeared for the modified adsorbent after As adsorption, which can be attributed to stretching vibrations of As-O [37].

### Applications and limitations of the functionalized diatom shells

The maximum arsenic adsorption capacity obtained from this study was 3.53 and 10.99 mg g^-1^for raw and modified adsorbent, respectively. The chemically modified adsorbent exhibits much higher sorption capacity than other sorbents (Table 4). The modified adsorbent is also more favorable for arsenic removal than activated alumina because of its slow sorption kinetics and low cost associated with this most commonly used sorbent [7]. The modified adsorbent holds promise for the removal of As(III) from water and other aqueous waste streams, thus eliminating the need for a prior-oxidation process that typically requires the use of oxidizing agents and catalysts.

**Table 4:** Comparison of arsenic adsorption capacity with other adsorbents.

Adsorbent	Maximum adsorption capacity (mg g^-1^)	References
Bacterial biomass	0.58	[6]
Activated alumina	3.48	[38]
Goethite	0.38	[39]
Hematite	0.26	[39]
Fe-Mn binary oxide modified diatomite	1.68	[40]
Polyaluminum granulate	18.00	[41]
Raw diatom silica shells	3.53	This study
Modified diatom silica shells	10.99	This study

The maximum adsorption capacity in this study was acquired under the experimental conditions at pH 4, so this sorbent can be directly used in acidic wastewater containing high concentration of arsenic [42]. For drinking water treatment, the low pH requirement may present a practical constraint unless prior pH adjustment is economically feasible. Surface functionalization could be further refined by considering limiting factors such as pH and sorbent regeneration. Future scale-up experiments are warranted using kinetic and thermodynamic parameters obtained from the present study. For example, the competition of DOM can be examined with real water/wastewater samples to test the practical use of this biosorbent in removing arsenic in the presence of DOM.

## Conclusions

With a primary goal of developing a cost-effective sorbent employing diatom frustules, this study characterized the adsorption properties and elucidated mechanisms responsible for the improved removal of arsenic. As(III) adsorption was best fitted with the Langmuir-Freundlich model. The mass transfer model indicated that both the film and intrapore diffusion can be the rate-determining step in the adsorption process. Possessing bifunctional (thiols and amino) characteristics following the chemical modifications, this unique sorbent proceeded through a chemisorption mechanism, which was verified by FTIR and XPS. Further spectroscopic evidence by SEM and FTIR indicated that the thiol and amino groups were grafted on the surface of the diatom frustules.

## Supporting Information

S1 Fig SEM images of the original (a, b, c), modified (d, e, f), and modified with As-sorbed diatom frustules (g, h, i). (TIF)

S2 Fig Nitrogen adsorption-desorption isotherms of raw (top) and modified samples (bottom). (TIF)

S3 Fig The points of zero charge (pH_zpc_) for the raw and modified adsorbent. (TIF)

S4 Fig Plot of lnC/C_0_ vs. time for the acquisition of kinetic parameters. (TIF)

S5 Fig Effects of contact time (a) and adsorbent concentration (b) on As adsorption by raw and functionalized diatom frustules. (TIF)

S1 Table Textural characteristics of the raw and functionalized diatom frustules. (DOCX)

S2 Table FTIR spectral characteristics of raw and modified diatom frustules before and after adsorption. (DOCX)

S3 Table External and internal mass transfer parameters for As adsorption of arsenite on modified diatom frustules. (DOCX)

S1 Text Additional experimental details, modeling and result explanations. (DOCX)
