# Supplementary figures and images for: Enhanced Adsorption of Trivalent Arsenic from Water by Functionalized Diatom Silica Shells
Source: PLoS One. 2015 Apr 2;10(4):e0123395. doi: 10.1371/journal.pone.0123395 (PMC4383452; doi:10.1371/journal.pone.0123395)

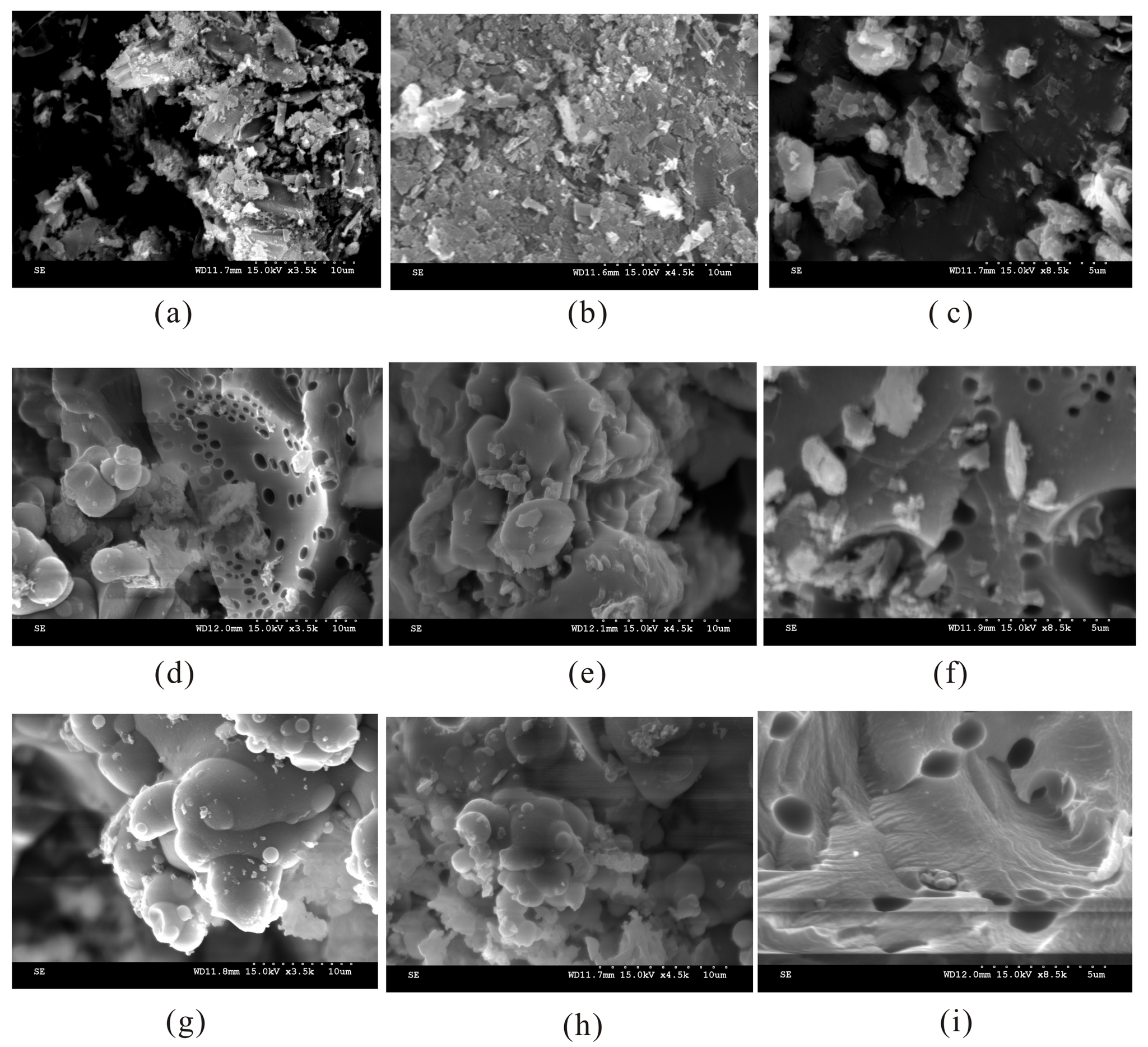

Supplement: S1 Fig — (TIF) [file pone.0123395.s001.tif]

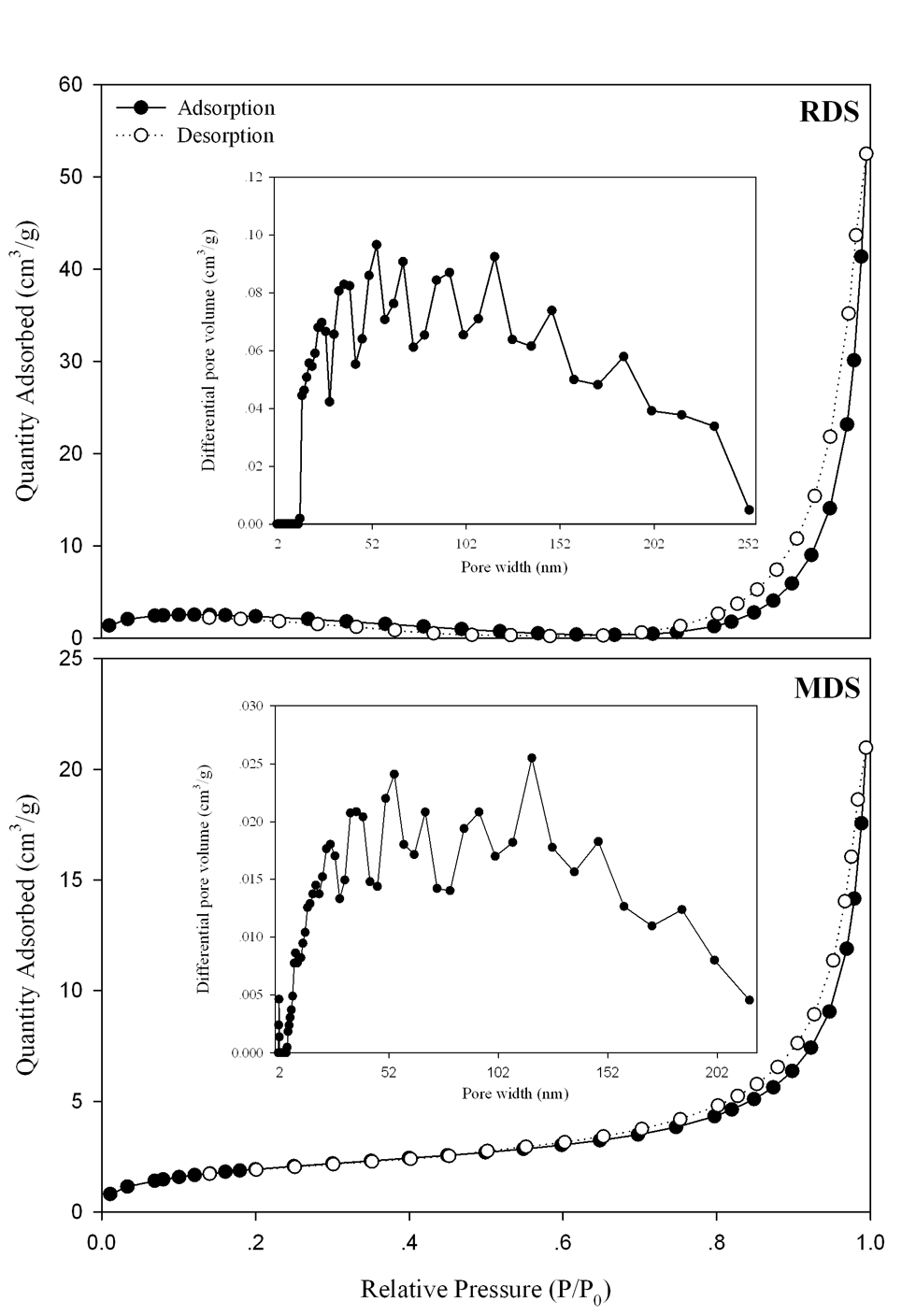

Supplement: S2 Fig — (TIF) [file pone.0123395.s002.tif]

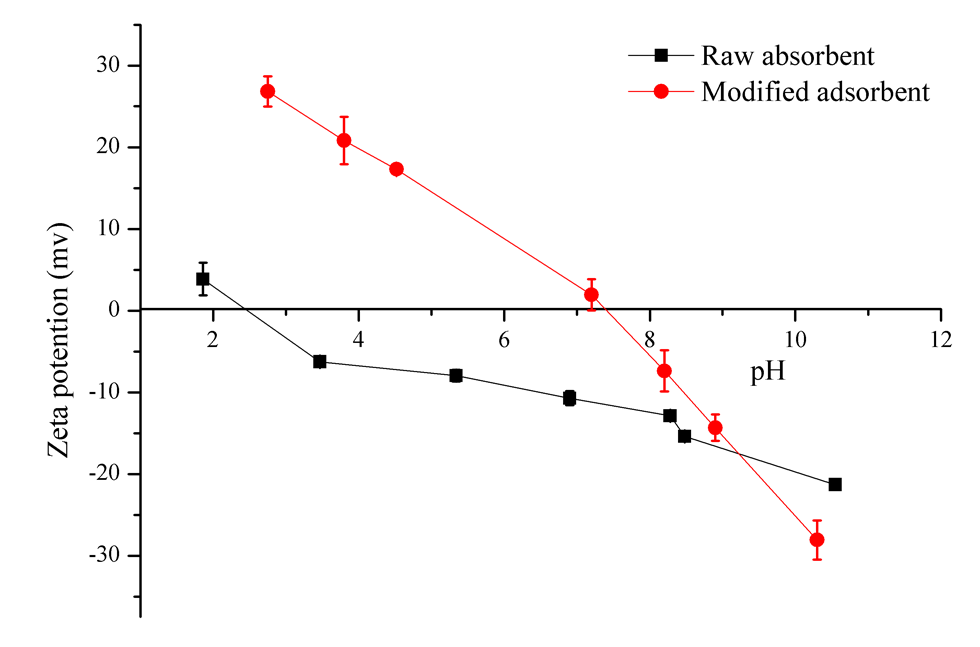

Supplement: S3 Fig — (TIF) [file pone.0123395.s003.tif]

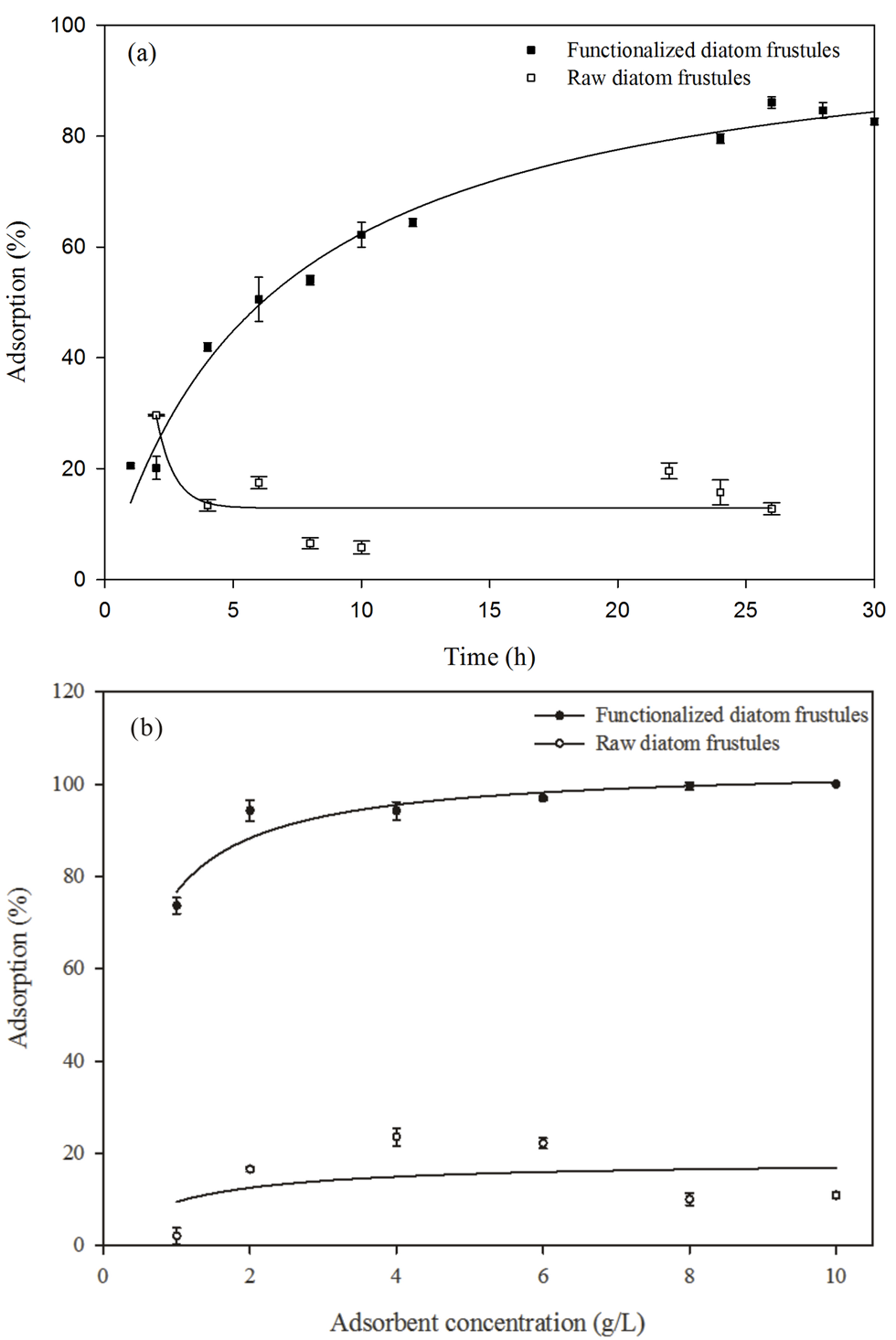

Supplement: S4 Fig — (TIF) [file pone.0123395.s004.tif]

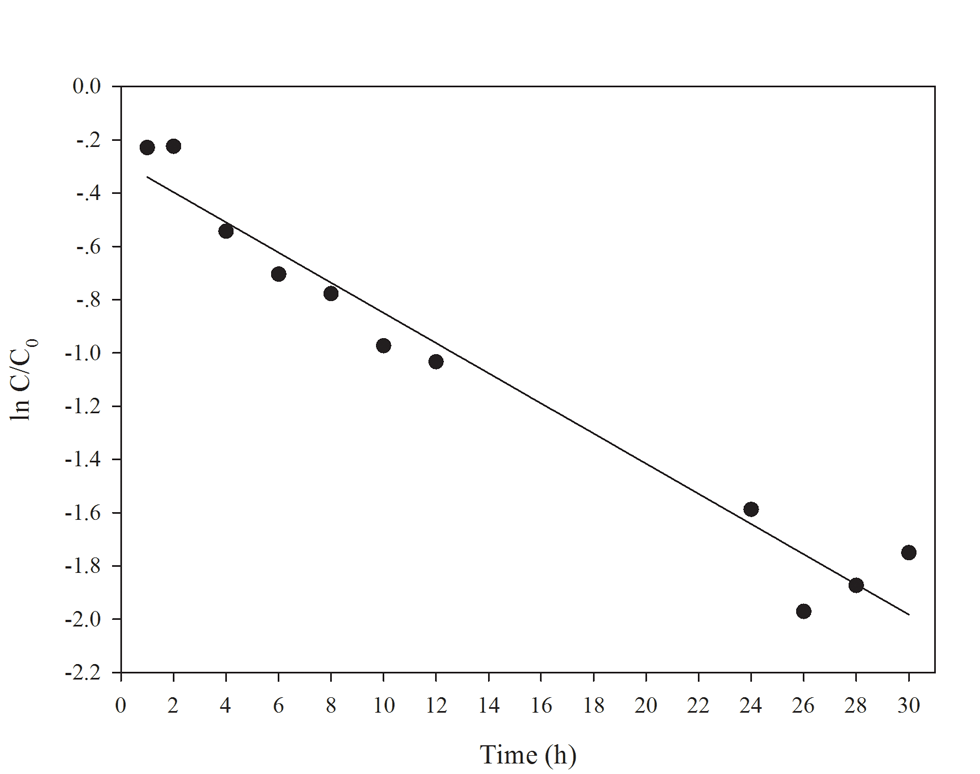

Supplement: S5 Fig — (TIF) [file pone.0123395.s005.tif]
