# Supplementary material for: Enhanced Adsorption of Trivalent Arsenic from Water by Functionalized Diatom Silica Shells
Source: PLoS One. 2015 Apr 2;10(4):e0123395. doi: 10.1371/journal.pone.0123395 (PMC4383452; doi:10.1371/journal.pone.0123395)
Supplement: S1 Table — (DOCX) [file pone.0123395.s006.docx]

**S1_** **Table** Textural characteristics of the raw and functionalized diatom frustules

| Samples | S_BET_ (m^2^/g) | V_T_ (cm^3^/g) | V_mi_ (cm^3^/g) | V_ext_(cm^3^/g) | r (μm) |
| --- | --- | --- | --- | --- | --- |
| RDS ^a^ | 7.87 ± 0.39 | 0.036 | 0.006 | 0.030 | 0.76 |
| MDS ^b^ | 7.30 ± 0.03 | 0.018 | 0.003 | 0.015 | 0.82 |

^a^ RDS = Raw diatom silica shell; ^b^ MDS = Modified diatom silica shell
