# Supplementary material for: Enhanced Adsorption of Trivalent Arsenic from Water by Functionalized Diatom Silica Shells
Source: PLoS One. 2015 Apr 2;10(4):e0123395. doi: 10.1371/journal.pone.0123395 (PMC4383452; doi:10.1371/journal.pone.0123395)
Supplement: S2 Table — (DOCX) [file pone.0123395.s007.docx]

**S2_Table FTIR spectral characteristics of raw and modified diatom frustules before and after adsorption**

| Wavenumber (cm^-1^) | | | Assignments |
| --- | --- | --- | --- |
| Raw adsorbent | Modified adsorbent | After adsorption |  |
| 3743 | 3673 | 3676 | O-H stretching of Si-OH |
| 3417 | 3363 | 3423 | O-H stretching of water, acid etc. |
| 2926 | 2929 | 2928 | Symmetric CH_2_ stretching vibration |
| - | 2571 | 2559 | Stretching vibrations of -SH |
| 1652 | - | - | C=O stretching; amide I |
| - | 1592 | 1619 | Amino bond |
| 1385 | 1411, 1384 | 1412 | C=O stretching; Carboxyl group |
| - | 1124 | 1131 | ≡C-N stretching |
| 1089 | 1029 | 1029 | C=O and Si-O stretching, plane deformation |
| 798, 561, 461 | 794, 692, 460 | 791, 691, 466 | Stretching vibrations of Si-O groups |
