# Supplementary material for: Enhanced Adsorption of Trivalent Arsenic from Water by Functionalized Diatom Silica Shells
Source: PLoS One. 2015 Apr 2;10(4):e0123395. doi: 10.1371/journal.pone.0123395 (PMC4383452; doi:10.1371/journal.pone.0123395)
Supplement: S3 Table — (DOCX) [file pone.0123395.s008.docx]

**S3_Table**  **External and internal mass transfer parameters for As adsorption on modified diatom frusutles**

| Modified Adsorbent | C_0_, mg L^-1^ | β_e_, m s^-1^ | a_s_, m^2^ kg^-1^ | ρ_p_, kg m^-3^ | d_p_, m | D_s_, m^2^ s^-1^ | r^2^ |
| --- | --- | --- | --- | --- | --- | --- | --- |
|  | 12 | 1.44×10^-6^ | 4.7×10^4^ | 156 | 8.2×10^-7^ | 7.2×10^-14^ | 0.963 |
