# Supplementary material for: Enhanced Adsorption of Trivalent Arsenic from Water by Functionalized Diatom Silica Shells
Source: PLoS One. 2015 Apr 2;10(4):e0123395. doi: 10.1371/journal.pone.0123395 (PMC4383452; doi:10.1371/journal.pone.0123395)

**Supporting Information**

**Materials and methods**

As(III) standard solution (10 mM) was prepared from NaAsO_2_. The pH values in the adsorption process were adjusted using several buffer solutions. Sodium phosphate buffer solutions in the range of pH 2-4 were prepared by adding an appropriate amount of phosphoric acid to sodium dihydrogen phosphate solution. Ammonium acetate buffers in the range of 4-6 were prepared by adding an appropriate amount of acetic acid to ammonium acetate solutions. Phosphate buffers with pH 6-8 were prepared by adding an appropriate amount of sodium hydroxide to potassium dihydrogen phosphate solution. Ammonium chloride buffer solutions with pH 9-11 were prepared by adding an appropriate amount of ammonia to ammonium chloride solutions.

The batch adsorption studies were conducted in triplicate under different experimental conditions: the contact times (1, 2, 4, 6, 8, 10, 12, 24, 26, 28, 30 h), pHs (2, 4, 5, 7, 9, 10), initial As concentrations (10, 20, 40, 80, 160, 200, 300 μM), the adsorbent concentrations (1, 2, 4, 6, 8, 10 g L^-1^) and the temperatures (25, 35, 45, 55 ^0^C). The percent adsorption (η) of As was calculated as follows:

$\eta(\%)= (\mathrm{C}_{0}-\mathrm{C})/ C_{0}\times100$ (1)

where *C*_0_ and *C* are the initial and final As concentrations (μM), respectively.

**Non-linear error functions**

Four non-linear error functions were examined to determine best-fit parameters of isotherm and kinetic models, including the hybrid fractional error function (HYBRID), Marquardt’s percent standard deviation (MPSD), the average relative error (ARE), and the root mean standard error (RMSE) [1,2]. The hybrid fractional error function (HYBRID) is as follows:

$\frac{100}{\text{p-n}}\sum_{i=1}^{p} [\frac{\left(q_{e,meas}-q_{e,calc}\right)_{i}^{2}}{q_{e,meas}}]_{i}$ (2)

where q_e, meas_ is the measured adsorption capacity, q_e,calc_ is the model calculated adsorption capacity, p is the number of data points, and n is the parameters within the isotherm equation. The Marquardt’s percent standard deviation (MPSD) is determined by:

$100\left(\sqrt{\frac{1}{p-n}}\sum_{i=1}^{p} \left[\frac{\left(q_{e,meas}-q_{e,calc}\right)_{i}^{2}}{q_{e,meas}}\right]_{i}\right)$ (3)

The average relative error (ARE) is calculated as:

$$\frac{100}{p}\sum_{i=1}^{p} \left|\frac{q_{e,calc}-q_{e,meas}}{q_{e,meas}}\right|_{i}$$

The root mean standard error (RMSE) is calculated as:

(4)

where X_i_ is the value of each measured datum and X_m_ is the modelled value. In all of the error methods it was assumed that both the liquid-phase concentration and the solid-phase concentration contribute equally to weighting the error criterion for the model solution procedure.

**Scanning electron microscopy characterization**

There are three magnifications of SEM images (3500×, 4500×, and 8500×) for all three types of diatom frustule samples (S1_Fig.). The SEM characterization depicts detailed surface information for the diatom frustules, and distinguishes the surface changes after the chemical modification and As adsorption. The SEM images of original diatom frustules fragments present a typical porous diatom structure (a and b in S1_Fig. ). As discussed in the manuscript, the addition of coupling agents can facilitate the aggregation of the original fragments, resulting in a more elongated surface and more visible porous structure (d in S1_Fig. ). It is likely that some pores on the surface are covered by the reactant of coupling agents (e in S1_Fig.). Subsequent to arsenic adsorption, the reduced pores can be seen (g and h in S1_Fig.). Under high resolutions (f and i in S1_Fig.), a comparison of sorbent before and after As adsorption suggests the presence of some aggregates filled in the pores, which may be the surface reaction products with arsenic.

**Nitrogen adsorption characterization**

N_2_ adsorption-desorption isotherms were measured on a Tristar 3020 analyzer (Micromeritics Co., USA) at liquid nitrogen temperature. The samples were outgassed at 373 K for 12 h at the degas port and then transferred to the analysis port to degas further for 6 h below a relative pressure of 0.01 before measurement. Porous parameters of the raw and modified samples are listed in S1_Table, according to the calculation method reported previously by Liu et al. [3].

As can be seen in S1_Table, the specific surface area (S_BET_) and the pore volumes including total pore volume (V_T_), micropore volume (V_mi_), and external pore volume (V_ext_), decreased after the chemical modification of diatom frustules, whereas the average particle size (r) of functionalized diatom frustules increased. These results suggest that physisorption could not be the main mechanism attributable to the improved arsenic adsorption.

The texture features (e.g., surface area and pore structure) of the raw and modified diatom frustules were analyzed by nitrogen adsorption-desorption methods. As shown in S2_Fig., the nitrogen sorption isotherms of the raw diatom frustules exhibited a typical type III pattern according to the IUPAC classification [4]. In contrast, the nitrogen sorption isotherms of the modified diatom frustules showed a typical type II pattern, indicating that the modification processes may enhance the interactions between adsorbent and adsorbate. According to the distribution of pore diameter (insert in S2_Fig.), although the distribution curve shows the mesopores at 2-50 nm in two types of adsorbents and small volumes of micropores at < 2 nm in functionalized diatom frustules, the macropores at > 50 nm occupied the majority of pores in the two adsorbents. These pore sizes coincide with the isotherm types detailed above.

**Fourier transform infrared spectroscopy (FTIR)**

The FTIR spectra of raw and modified diatom frustules and As-loaded adsorbent were taken by AVARAT-370 model spectrophotometer in order to obtain information on the nature of functionalized diatom frustules and probable interactions between the functional groups on the diatom frustule surface and arsenic (S2_Table). The spectra information for some common functional groups in this work is presented as follows. The weak bands at 3743 and 3673 cm^-1^ can be assigned to the stretching of Si-OH groups [5], whereas the bands at 3417 and 3363 cm^-1^ are attributed to the stretching of O-H groups derived from physiosorbed water and inter- or intramolecular hydrogen bonding of polymeric compounds such as carboxylic acids [6, 7]. The vibrations at 2926 and 2929 cm^-1^ are associated with the asymmetric and symmetric stretching modes of the –CH_2_– moiety related to the carbon chain of organosilane molecules in the modified diatom samples. The bands typical to the stretching vibrations of oriented Si-O-Si bonds and Si-O groups are observed at 1089, 798, 461 cm^-1^ in raw diatom frustules, whereas the bands are shifted to 1029, 794 460 cm^-1^ through the chemical modification. After adsorption, the O-H stretching vibration of Si-OH groups is shifted to 3676 cm^-1^, and the stretching vibrations of Si-O groups are shifted to 466 cm^-1^, indicating the involvement of Si-OH during the adsorption of arsenic.

For the functional groups that were introduced on the surface of diatom frustules, the peaks at 1652 cm^-1^ of raw diatom frustules related to amide I (randomly coiled and α-helix) were observed [8]. However, the peaks disappeared in the modified samples and the amino bonds (–NH_2_) at 1592 cm^-1^ may have contributed to the scissoring vibration of the –NH_2_ terminal group [9]. On the other hand, the –SH groups were only observed in the modified samples with the stretching vibration at about 2571 cm^-1^[10]. Therefore, the presence of –NH_2_ and –SH functional groups on the modified diatom surface clearly indicates the effectiveness of APTMS and MPTMS to achieve the functionalized silica surface. After adsorption, the stretching bands of the –NH_2_ groups are shifted to 1619 cm^-1^, and the stretching bands of –SH group are shifted to 2559 cm^-1^ with a marked change in the transmittance. These shifts in the wave number of specific bands indicate that these introduced functional groups (–NH_2_ and –SH) are mainly involved in the adsorption of arsenic.

**Effects of contact time and adsorbent concentration**

The effects of contact time on arsenic adsorption were studied under the conditions of 160 μM As, 1 g L^-1^ biomass and pH 4. As seen from a in S5_Fig., the raw diatom frustules exhibited poor adsorption throughout the duration of the experiments, whereas increased adsorption was observed with the increasing contact time for the modified sorbent. For the modified diatom frustules, equilibrium reached at 26 h with 86% As removal efficiency, indicating the fast sorption and high efficiency of the biosorbent. In regard to the effects of various adsorbent concentrations, b in S5 Fig. shows a markedly increased adsorption efficiency with the increasing adsorbent concentration up to 2 g L^-1^ in the functionalized diatom frustules. At adsorbent concentration of 2 g L^-1^ and 10 g L^-1^, the adsorption efficiency reached as high as 94.29% and 99.99%, respectively.

**Adsorption isotherms of arsenic on the functionalized diatom frustules**

The amount adsorbed increased markedly with the increasing arsenic equilibrium concentrations in solution. Such an increase may be attributed to the active functional groups on the adsorbent surface, such as the introduced –SH and –NH. The small molar ratios of adsorbed As to -SH or –NH in the range of 1.68×10^-4^ to 5.57×10^-3^ was obtained by theoretical calculations, assuming all the mercaptopropyl silica synthesized from MPTMS and APTES has been introduced onto diatom frustules. This small molar ratio implies that most -SH and –NH functional groups were inactive on the adsorbent surface [11]. An increased arsenic concentration could potentially activate the functional groups on the adsorbent surface, thus enhancing their interactions with the adsorbent surface.

**Adsorption kinetic modeling**

The first governing equation describing external mass transfer from bulk liquid to the surface of adsorbents is based on Fick’s first law [12]:

$N_{L}= -D_{1} \nabla C = -\beta_{e} (C - C_{I})$ (5)

where *N*_L_ is the mass transfer rate per unit surface area, *D*_1_ is the free diffusivity for As(III), *C* and *C*_I_ are the bulk concentration and concentration on the external surface of adsorbent, respectively (mol m^-3^), $\beta_{e}$is the external mass transfer coefficient (m s^-1^). Assuming negligible *C*_I_ at the beginning of the sorption time, Eq. 6 can be simplified after integration [12]:

$\ln\frac{C}{C_{0}}=-\beta_{e}\frac{Ma_{s}}{V_{L}}t$ (6)

where *M* is the mass of adsorbent (kg), *V*_L_ is the solution volume (dm^3^), $a_{s}$is the specific external surface area (m^2^ kg^-1^) as defined by:

$a_{s}= 6/(\rho_{p}d_{p})$ (7)

where $\rho_{p}$is apparent particle density (156 kg m^-3^) and $d_{p}$is mean particle size (8.2×10^-7^m). Using the slope of linear regression (slope =−0.0566, r^2^ = 0.963) between $\ln(C/C_{0})$and t (S5_ Fig.) and the values of M (1 kg), V_L_ (1 L) and a_s_ (4.7×10^4^ m^2^ kg^-1^), the $\beta_{e}$value is estimated to be 1.44×10^-6^ m s^-1^ for As(III).

The second governing equation describing arsenite diffusion in the pores of diatom silica shells is Eq. (8) below with initial and boundary equations detailed in [12]:

$\frac{\partial q}{\partial t}=D_{s}\frac{\partial^{2}q}{\partial r^{2}}$ (8)

where $\varepsilon_{p}$is the particle porosity, C_p_ is arsenite concentration in the pores at r = d_p_/2, q is the arsenite concentration in solid phase (mol kg^-1^), D_p_ is the internal (intrapore) diffusion coefficient (m^2^ s^-1^), which is directly proportional to the free diffusivity of arsenic and two parameters related to the porous structure of diatom frustules that impacts arsenic diffusion in pore water as follows [13]:

$D_{p}=\frac{\varepsilon_{p}D_{1}}{\tau}$ (9)

where D_1_ is the free diffusivity for As(III) (11.6×10^-10^ m^2^ s^-1^) [13], the particle porosity ($\varepsilon_{p}$) can be determined from the BET analysis ($\varepsilon_{p}$= 0.17), and $\tau$is the tortuosity factor which can be calculated from $\varepsilon_{p}$according to: $\tau= (2-\varepsilon_{p})^{2}/\varepsilon_{p}$. Using values of $\varepsilon_{p}$(0.17) and $\tau$(19.7), the D_p_ is estimated to be 1.0×10^-11^ m^2^ s^-1^.

**References**

[1] Allen SJ, Mckay G, Porter JF (2004) Adsorption isotherm models for basic dye adsorption by peat in single and binary component systems. J Colloid Interface Sci 280: 322-333.

[2] Garcia-Torres L, Caballero-Novella JJ, Gomez-Candon D, De-Castro AI (2014) Semi-automatic normalization of multitemporal remote images based on vegerative pseudo-invariant features PLOS One 9: 91275-91284.

[3] Liu QS, Zheng T, Li N, Wang P, Abulikemu G (2010) Modification of bamboo-based activated carbon using microwave radiation and its effects on the adsorption of methylene blue. Appl Surf Sci 256*:* 3309-3315.

[4] Rouquerol F, Rouquerol J, Sing K (1999) Adsorption by powders and porous solids. Academic Press: London, 27-50.

[5] Yuan P, Liu D, Tan DY, Liu KK, Yu HG, et al. (2013) Surface silylation of mesoporous/macroporous diatomite (diatomaceous earth) and its function in Cu(II) adsorption: The effects of heating pretreatment. Microporous Mesoporous Mater 170: 9-19.

[6] Iqbal M, Saeed A, Zafar SI (2009) FTIR spectrophotometry, kinetics and adsorption isotherms modeling, ion exchange, and EDX analysis for understanding the mechanism of Cd^2+^ and Pb^2+^ removal by mango peel waste. J Hazard Mater 164: 161-171.

[7] Sari A, Citak D, Tuzen M (2010) Equilibrium, thermodynamic and kinetic studies on adsorption of Sb(III) from aqueous solution using low-cost natural diatomite. Chem Eng J 162:521-527.

[8] Yu Y, Addai-Mensah J, Losic D (2012) Functionalized diatom silica microparticles for removal of mercury ions. Sci Technol Adv Mater13:1-11.

[9] Heredia A, Figueira E, Rodrigues CT, Rodríguez-Galván A, Basiuk VA, et al. (2012) Cd^2+^ affects the growth, hierarchical structure and peptide composition of the biosilica of the freshwater diatom *Nitzschia palea* (Kützing) W. Smith. Phycol Res 60:229-240.

[10] Liang XF,Xu YM,Sun GH, Wang L, Sun Y, et al. (2009) Preparation, characterization of thiol-functionalized silica and application for sorption of Pb^2+^ and Cd^2+^. Colloid Surf Physicochem Eng Aspect 349:61-68.

[11] Hao JM, Han MJ, Meng XG (2009) Preparation and evaluation of thiol-functionalized activated alumina for arsenite removal from water. J Hazard Mater 167: 1215-1221.

[12] Chang CF, Lee SC (2012) Adsorption behavior of pesticide methomyl on activated carbon in a high gravity rotating packed bed reactor. Water Res 46:2869-2880.

[13] Cui H, Su Y, Li Q, Gao SA, Shang JK (2013) Exceptional arsenic (III,V) removal performance of highly porous, nanostructured ZrO_2_ spheres for ﬁxed bed reactors and the full-scale system modeling. Water Res 47:6258-6268.

**Graphic Abstract:**


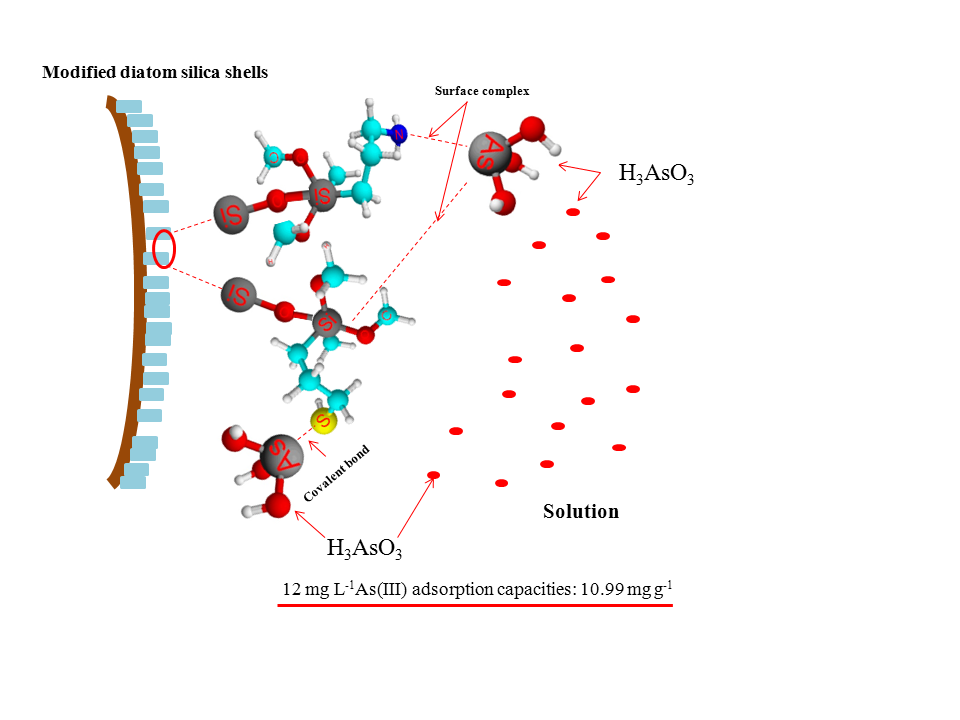

Supplement: S1 Text — (DOCX) [file pone.0123395.s009.docx]
